# Oral Temperature and pH Influence Dietary Nitrate Metabolism in Healthy Adults

**DOI:** 10.3390/nu15030784

**Published:** 2023-02-03

**Authors:** Stuart P. Cocksedge, Adam J. Causer, Paul G. Winyard, Andrew M. Jones, Stephen J. Bailey

**Affiliations:** 1 School of Sport, Exercise and Health Sciences, Loughborough University, Loughborough LE11 3TU, UK; 2 Sport and Health Sciences, University of Exeter, Exeter EX1 2LU, UK; 3 Exeter Medical School, University of Exeter, Exeter EX1 2LU, UK

**Keywords:** entero-salivary circulation, nitrite, nitric oxide, nutrition

## Abstract

This study tested the hypothesis that the increases in salivary and plasma [NO_2_^−^] after dietary NO_3_^−^ supplementation would be greater when oral temperature and pH were independently elevated, and increased further when oral temperature and pH were elevated concurrently. Seven healthy males (mean ± SD, age 23 ± 4 years) ingested 70 mL of beetroot juice concentrate (BR, which provided ~6.2 mmol NO_3_^−^) during six separate laboratory visits. In a randomised crossover experimental design, salivary and plasma [NO_3_^−^] and [NO_2_^−^] were assessed at a neutral oral pH with a low (T_Lo_-pH_Norm_), intermediate (T_Mid_-pH_Norm_), and high (T_Hi_-pH_Norm_) oral temperature, and when the oral pH was increased at a low (T_Lo_-pH_Hi_), intermediate (T_Mid_-pH_Hi_), and high (T_Hi_-pH_Hi_) oral temperature. Compared with the T_Mid_-pH_Norm_ condition (976 ± 388 µM), the mean salivary [NO_2_^−^] 1–3 h post BR ingestion was higher in the T_Mid_-pH_Hi_ (1855 ± 423 µM), T_Hi_-pH_Norm_ (1371 ± 653 µM), T_Hi_-pH_Hi_ (1792 ± 741 µM), T_Lo_-pH_Norm_ (1495 ± 502 µM), and T_Lo_-pH_Hi_ (2013 ± 662 µM) conditions, with salivary [NO_2_^−^] also higher at a given oral temperature when the oral pH was increased (*p* < 0.05). Plasma [NO_2_^−^] was higher 3 h post BR ingestion in the T_Mid_-pH_Hi_, T_Hi_-pH_Hi_, and T_Lo_-pH_Hi_ conditions, but not the T_Lo_-pH_Norm_ and T_Hi_-pH_Norm_ conditions, compared with T_Mid_-pH_Norm_ (*p* < 0.05). Therefore, despite ingesting the same NO_3_^−^ dose, the increases in salivary [NO_2_^−^] varied depending on the temperature and pH of the oral cavity, while the plasma [NO_2_^−^] increased independently of oral temperature, but to a greater extent at a higher oral pH.

## 1. Introduction

The inorganic anions, nitrate (NO_3_^−^) and nitrite (NO_2_^−^), have historically been considered as adverse carcinogenic agents or to be inert derivatives of nitric oxide (NO) oxidation [1]. However, more recent data indicate that NO_3_^−^ and NO_2_^−^ can be recycled back to the multifaceted physiological signalling molecule, NO, and that dietary NO_3_^−^ supplementation can enhance aspects of health and exercise performance [2,3]. While ~60% of ingested NO_3_^−^ is excreted in the urine [4], ~25% is extracted from the circulation by the salivary glands [5], via the NO_3_^−^/H^+^ cotransporter, sialin [6]. NO_3_^−^ is concentrated within, and subsequently excreted by, the salivary glands [7] for subsequent reduction to NO_2_^−^ by certain taxa of the oral microflora [8,9,10]. NO_2_^−^-rich saliva is then swallowed and subsequently reduced to NO and various reactive nitrogen intermediates within the stomach [2,11], but it is also clear that circulating plasma [NO_2_^−^] is increased post NO_3_^−^ supplementation [7,12]. This circulating plasma NO_2_^−^ can undergo a one-electron reduction to NO, in a reaction catalysed by numerous NO_2_^−^ reductases [13,14], with improvements in cardiovascular health markers and exercise responses positively associated with oral NO_3_^−^ reduction and the increase in plasma [NO_2_^−^] after NO_3_^−^ supplementation [13,15,16,17]. Therefore, enhancing dietary NO_3_^−^ metabolism has the potential to augment NO synthesis and to improve various aspects of human health and function.

There is evidence that mammalian tissues have the capacity to directly reduce NO_3_^−^ to NO_2_^−^ via xanthine oxidoreductase [18,19], in addition to NO_3_^−^ reduction catalysed by the oral microbiome [8,9,10]. However, it has been suggested that humans have a greater proportional dependence on NO_3_^−^ reduction via the oral microbiome than by xanthine oxidoreductase compared with some other mammals [18]. As such, oral NO_3_^−^ reduction is integral to NO_3_^−^ metabolism and its accompanying physiological effects [20]. Indeed, the administration of chlorohexidine-containing mouthwash, which transiently eradicates oral bacteria, essentially abolishes the increases in salivary and plasma [NO_2_^−^] and the alterations in vascular function and exercise capacity that ensue after NO_3_^−^ ingestion [21,22,23,24,25]. There is also evidence that oral NO_3_^−^ reduction to NO_2_^−^ is independently influenced by oral pH and environmental temperature. Specifically, oral NO_3_^−^ reduction is enhanced with increasing the pH, with peak oral NO_3_^−^ reduction suggested to occur at pH 8 [26,27,28], and by an increase in oral temperature, as inferred by the greater oral NO_3_^−^ reduction in summer months, compared with autumn or winter months [26,28]. However, oral temperature and pH were not directly determined in these previous studies, and it is unclear whether combining elevated oral temperature and pH elicits an additive effect on oral NO_3_^−^ reduction. In addition, the extent to which potential changes in oral NO_3_^−^ reduction with altered oral temperature and pH influences plasma [NO_2_^−^] after dietary NO_3_^−^ supplementation has yet to be investigated. This is important in order to improve our understanding of the factors that can influence dietary NO_3_^−^ metabolism in healthy humans, with potential implications for the physiological and performance effects afforded by dietary NO_3_^−^ supplementation. 

The purpose of the present study was to assess the independent and combined effects of altering oral temperature and pH on the salivary and plasma [NO_2_^−^] after ingesting a fixed NO_3_^−^ bolus in healthy adults. It was hypothesised that increasing the oral temperature and pH would independently and additively increase the salivary and plasma [NO_2_^−^] after dietary NO_3_^−^ supplementation.

## 2. Materials and Methods

### 2.1. Participant Characteristics

Seven males (mean ± SD, age 23 ± 4 years, height 1.79 ± 0.06 m, and body mass 76 ± 11 kg) were recruited from the university student community to participate in this study. All procedures employed in this study were approved by the Institutional Research Ethics Committee. Participants gave their written informed consent to participate prior to the commencement of the study, after the experimental procedures, associated risks, and potential benefits of participation had been explained. Participants were instructed to arrive at each laboratory testing session in a rested state after an overnight fast. As the reduction of NO_3_^−^ to NO_2_^−^ in the oral cavity is thwarted by antibacterial mouthwash [23], participants were required to refrain from mouthwash use for the duration of the study. Similarly, as cigarette smokers exhibit impaired salivary NO_3_^−^ uptake and NO_3_^−^ metabolism after dietary NO_3_^−^ supplementation compared with non-smokers, non-smoking participants were recruited to participate in the current study [29]. Each participant was also asked to avoid the consumption of NO_3_^−^-rich, iodide-rich [30], and glucosinolate/thiocyante-rich [31] foods for 48 h, and from caffeine and alcohol ingestion 12 and 24 h before each test, respectively. All participants were instructed to maintain their habitual physical activity pattern for the duration of the study, and to avoid strenuous exercise in the 24 h preceding the testing sessions. All tests were performed at the same time of day (±1 h) in an air-conditioned laboratory at 20 °C.

### 2.2. Experimental Design

Participants were required to report to the laboratory on seven occasions over a 4–7-week period to complete the experimental testing. On the first visit to the laboratory, participants were familiarized with all procedures used in the study, including venous cannulation, saliva sampling, oral temperature assessment, and mouth rinse administration (see below). For the remaining six visits, participants ingested a 70 mL beetroot juice concentrate (BR), which contained ~6.2 mmol NO_3_^−^, and the temperature and pH of the oral cavity were manipulated to assess the effect of these variables on dietary NO_3_^−^ metabolism. Specifically, salivary and plasma [NO_3_^−^] and [NO_2_^−^] were assessed pre and post BR ingestion in the following experimental conditions: low oral temperature, neutral oral pH (T_Lo_-pH_Norm_); low oral temperature, high oral pH (T_Lo_-pH_Hi_); intermediate oral temperature, neutral pH (T_Mid_-pH_Norm_); intermediate oral temperature, high oral pH (T_Mid_-pH_Hi_); high oral temperature, neutral oral pH (T_Hi_-pH_Norm_); and high oral temperature and pH (T_Hi_-pH_Hi_). These experimental conditions were administered in a randomized crossover experimental design.

### 2.3. Data Collection Procedures

Participants arrived at the laboratory in the morning and were provided with a standardized breakfast of 54 g of porridge oats (Oats So Simple, Quaker Oats) prepared with 180 mL of tap water and one 20 g sachet of golden syrup (Lyle’s golden syrup). A cannula (Insyte-W ^TM^ Becton-Dickinson, Madrid, Spain) was subsequently inserted into a forearm vein and a baseline blood sample was drawn into a 6 mL lithium-heparin vacutainer (Sarstedt, Leicester, UK). Blood samples were centrifuged at 3500× *g* and 4 °C for 10 min, within 1 min of collection. The plasma was subsequently extracted and immediately frozen at −80 °C for later analysis of [NO_3_^−^] and [NO_2_^−^]. The cannula was kept patent through the infusion of 0.9% saline at a rate of 10 mL·h^−^^1^ for the duration of the protocol. An unstimulated saliva sample (~1.2 mL) was then obtained and immediately analysed for salivary pH using a S_I_ series pH meter (Sentron, Leek, The Netherlands), which was calibrated prior to each test according to the manufacturer’s instructions. Saliva was subsequently frozen at −80 °C for later analysis of [NO_3_^−^] and [NO_2_^−^]. Baseline oral cavity temperature was measured for 10 min by placing a temperature probe (Carefusion, IL, USA) under the tongue with a closed mouth and with participants breathing through their nose. Data were captured at 0.33 Hz via a Squirrel SQ2020 Series Data Logger (Grant, Cambridgeshire, UK). Participants then consumed a 70 mL BR bolus, and blood and saliva were sampled hourly for 3 h post BR ingestion and processed as described above.

After BR ingestion, the temperature of the oral cavity was measured for a further 5 min with a closed mouth and nasal breathing in the Temp_Mid_-pH_Norm_ and Temp_Mid_-pH_Hi_ conditions, or with an open mouth with the participants breathing only through the mouth in the Temp_Lo_-pH_Norm_ and Temp_Lo_-pH_Hi_ conditions. In the Temp_Hi_-pH_Norm_ and Temp_Hi_-pH_Hi_ conditions, participants breathed through their nose with a closed mouth and two fleeced, neck hot water bottles containing boiling water were applied anti-parallel. Specifically, the apex of the inferior hot water bottle was in contact with the posterior surface of the neck, with the apex of the superior hot water bottle in contact with the anterior surface of the neck. The superior surface of the upper hot water bottle rested on the submandibular space. In addition, a heated gel pack (Reliance Medical, Cheshire, UK), which was heated for 45 s in a microwave oven, was applied to the left cheek and held in place with an elastic Velcro strap. Participants adopted these respective breathing patterns and mouth positions for 2.5 h. In all tests, participants employed nasal breathing with a closed mouth for the remaining 30 min of the condition. Hot water bottles and gel packs were replaced every 30 min up to 2.5 h to maintain the elevated oral cavity temperature based on the data obtained from pilot testing in the Temp_Hi_-pH_Norm_ and Temp_Hi_-pH_Hi_ conditions. The temperature of the oral cavity was measured for 10 min at 30 min intervals throughout the protocol.

Following oral temperature assessment, participants mouth rinsed with a 30 mL solution for 2 min. Participants were instructed to swallow any saliva in their mouth prior to the mouth rinse, to avoid swallowing the mouth rinse, and to spit the mouth rinse into a beaker when instructed after 2 min had elapsed. In the Temp_Mid_-pH_Norm_, Temp_Hi_-pH_Norm_, and Temp_Lo_-pH_Norm_ conditions, participants rinsed with 30 mL tap water (pH 7.3 ± 0.1) while participants rinsed with a 30 mL 4.2 M NaHO_3_ solution (pH 8.1 ± 0.1) in the Temp_Mid_-pH_Hi_, Temp_Hi_-pH_Hi_, and Temp_Lo_-pH_Hi_ conditions. In the Temp_Mid_-pH_Norm_ and Temp_Mid_-pH_Hi_ conditions, mouth rinses were heated to 35.0 ± 1.2 °C in an attempt to maintain oral temperature, whereas mouth rinses were administered at room temperature (20.7 ± 0.1 °C) in the Temp_Lo_-pH_Norm_ and Temp_Lo_-pH_Hi_ conditions in an attempt to lower oral temperature, and heated to 40.4 ± 0.7 °C in an attempt to increase oral temperature in the Temp_Hi_-pH_Norm_ and Temp_Hi_-pH_Hi_ conditions. Mouth rinses were administered every 7.5 min based on pilot testing, which revealed that this provided a mean salivary pH of ~8 over this timeframe in the pH_HI_ conditions. Mouth rinsing ceased 2.5 h post BR ingestion.

Oral temperature at each time point was taken as the mean temperature over the final 5 min of collection, with the overall oral temperature for each experimental condition taken as the mean oral temperature over the first 2.5 h of the protocol. Overall salivary pH was taken as the mean salivary pH at the start of the protocol and 1 and 2 h post BR consumption.

### 2.4. [NO_2_^−^] and [NO_3_^−^] Analysis

Salivary and plasma [NO_2_^−^] and [NO_3_^−^] were analysed as described previously [29,30,31]. Prior to [NO_2_^−^] and [NO_3_^−^] analysis, all glassware, utensils, and surfaces were rinsed with deionized water to remove residual NO intermediates. Plasma samples were deproteinized using zinc sulfate (ZnSO_4_)/sodium hydroxide (NaOH) precipitation prior to the determination of [NO_3_^−^]. Firstly, 500 μL of 0.18 N NaOH was added to 100 µL of sample followed by 5 min incubation at room temperature. Subsequently, samples were treated with 300 μL aqueous ZnSO_4_ (5% *w*/*v*) and vortexed for 30 s before undergoing an additional 10 min incubation period at room temperature. The samples were then centrifuged at 3500× *g* for 5 min, and the supernatant was removed for subsequent analysis. The [NO_3_^−^] of the deproteinized plasma sample was determined by its reduction to NO in the presence of 0.8 % (*w*/*v*) vanadium chloride in 1 M hydrochloric acid within an air-tight purging vessel. Plasma samples were introduced to the vessel via 50 uL injections into the septum at the top of the vessel. The spectral emission of electronically excited nitrogen dioxide, derived from the reaction of NO with ozone, was detected by a thermoelectrically cooled, red-sensitive photomultiplier tube housed in a Sievers gas-phase chemiluminescence nitric oxide analyser (Sievers NOA 280i. Analytix Ltd., Durham, UK). The [NO_3_^−^] was determined by plotting the signal (mV) area against a calibration plot of sodium nitrate standards. The [NO_2_^−^] of the undiluted (non-deproteinized) plasma was determined by its reduction to NO in the presence of glacial acetic acid and aqueous sodium iodide (4% *w*/*v*), with the calibration performed using sodium nitrite standards. Then, 100 uL injections were used for the plasma [NO_2_^−^] determination. After thawing at room temperature, the saliva samples were centrifuged for 10 min at 17,000× *g* and the supernatant was removed for subsequent analysis. The supernatant was diluted 100-fold with deionized water and [NO_3_^−^] and [NO_2_^−^] were determined from 50 uL injections using the same reagents describe above for the plasma analyses.

### 2.5. Statistics

A two-way (condition × time) repeated measures ANOVA was employed to determine the independent and combined effects of manipulating oral temperature and pH on salivary and plasma [NO_3_^−^] and [NO_2_^−^]. A one-way repeated measures ANOVA was employed to determine the effects of oral temperature and pH manipulation on the mean salivary and plasma [NO_3_^−^] and [NO_2_^−^], oral temperature, and salivary pH across the experimental conditions. Where the analysis revealed a significant main or interaction effect, Fishers Least Significant Difference tests were employed to determine the origin of such effects. All data are presented as mean ± SD, unless otherwise indicated. Statistical significance was accepted when *p* < 0.05.

## 3. Results

### 3.1. Oral Temperature

**Figure 1:**
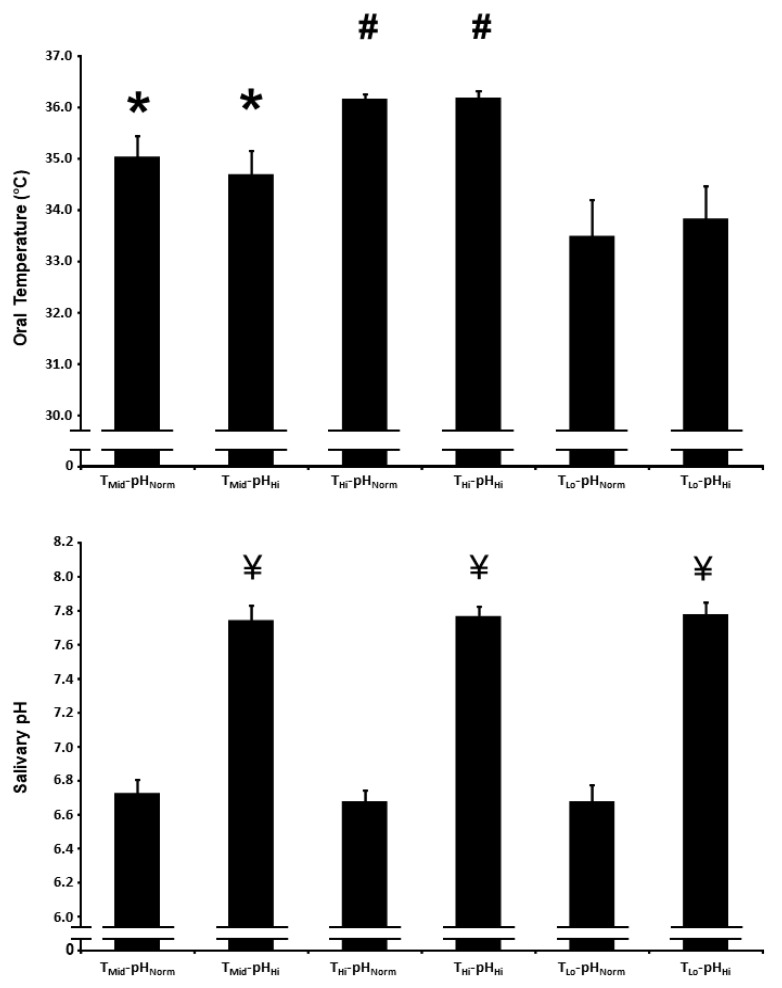
Oral temperature (upper panel) and salivary pH (lower panel) after acute nitrate-rich beetroot juice ingestion in intermediate temperature−neutral pH (T_Mid_-pH_Norm_), intermediate temperature−high pH (T_Mid_-pH_Hi_), high temperature−neutral pH (T_Hi_-pH_Norm_), high temperature and pH (T_Hi_-pH_Hi_), low temperature−neutral pH (T_Lo_-pH_Norm_), and low temperature−high pH (T_Lo_-pH_Hi_) conditions. The oral temperature data are expressed as the mean responses over the first 2.5 h of the protocol, while the salivary pH data are expressed as the mean responses over the first 2 h of the protocol. The filled bars represent the group mean ± SEM responses. * indicates higher than T_Lo_-pH_Norm_ and T_Lo_-pH_Hi_ (*p* < 0.05). # indicates higher than T_Mid_-pH_Norm_, T_Mid_-pH_Hi_, T_Lo_-pH_Norm_ and T_Lo_-pH_Hi_ (*p* < 0.05). ¥ indicates higher than T_Mid_-pH_Norm_, T_Hi_-pH_Norm_, and T_Lo_-pH_Norm_ (*p* < 0.05).

The oral temperature over the first 2.5 h of the protocol is presented in Figure 1. There was a main effect for the condition on oral temperature (*p* < 0.01). Oral temperature was higher in the T_Mid_-pH_Norm_ and T_Mid_-pH_Hi_ conditions compared with the T_Lo_-pH_Norm_ and T_Lo_-pH_Hi_ conditions, and higher in the T_Hi_-pH_Norm_ and T_Hi_-pH_Hi_ conditions compared with the T_Mid_-pH_Norm_, T_Mid_-pH_Hi_, T_Lo_-pH_Norm_, and T_Lo_-pH_Hi_ conditions (*p* < 0.01 for all comparisons). The mean oral temperature in the high, intermediate, and low temperature conditions was 36.2 ± 0.3 °C, 34.9 ± 1.2 °C, and 33.6 ± 1.7 °C, respectively.

### 3.2. Salivary pH

The salivary pH over the first 2 h of the protocol is presented in Figure 1. There was a main effect for condition on salivary pH (*p* < 0.01). Salivary pH was higher in the T_Mid_-pH_Hi_, T_Hi_-pH_Hi_, and T_Lo_-pH_Hi_ conditions compared with the T_Mid_-pH_Norm_, T_Hi_-pH_Norm_, and T_Lo_-pH_Norm_ conditions (*p* < 0.01 for all comparisons). The mean salivary pH in the high and normal conditions was 7.8 ± 0.2 and 6.7 ± 0.2, respectively.

### 3.3. Salivary [NO_3_^−^]

**Figure 2:**
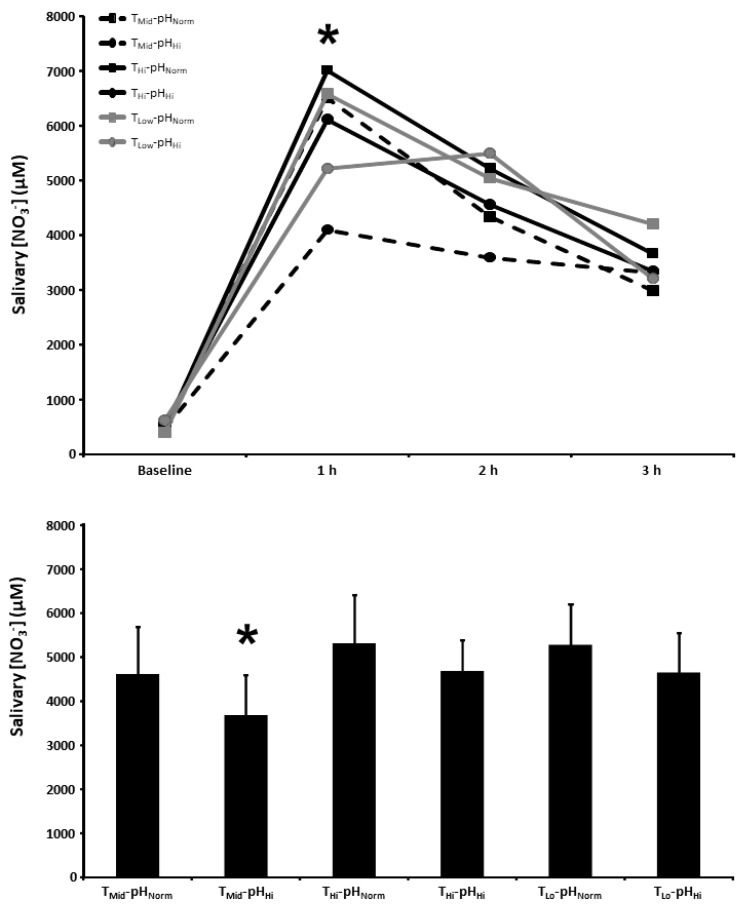
Salivary nitrate concentration ([NO_3_^−^]) time-course (upper panel) and the mean salivary [NO_3_^−^] at 1–3 h of the protocol (lower panel) after acute nitrate-rich beetroot juice ingestion in intermediate temperature−neutral pH (T_Mid_-pH_Norm_), intermediate temperature−high pH (T_Mid_-pH_Hi_), high temperature−neutral pH (T_Hi_-pH_Norm_), high temperature and pH (T_Hi_-pH_Hi_), low temperature−neutral pH (T_Lo_-pH_Norm_), and low temperature−high pH (T_Lo_-pH_Lo_) conditions. Salivary [NO_3_^−^] values across the protocol (upper panel) are expressed as group mean values with error bars omitted for clarity. The filled bars represent the group mean ± SEM responses in each experimental condition for mean salivary [NO_3_^−^] at 1–3 h of the protocol (lower panel). * indicates T_Mid_-pH_Hi_ lower than T_Hi_-pH_Norm_, T_Hi_-pH_Hi_, and T_Lo_-pH_Norm_ (*p* < 0.05).

The salivary [NO_3_^−^] at baseline and 1, 2, and 3 h post BR ingestion and the mean salivary [NO_3_^−^] 1–3 h post BR ingestion are presented in Figure 2. There was a main effect for time (*p* < 0.001), with salivary [NO_3_^−^] increasing above baseline in all experimental conditions. Salivary [NO_3_^−^] was lower in the T_Mid_-pH_Hi_ condition compared with the T_Hi_-pH_Norm_, T_Hi_-pH_Hi_, and T_Lo_-pH_Norm_ conditions 1 h following BR ingestion (*p* < 0.05). The mean salivary [NO_3_^−^] 1–3 h post BR ingestion was also lower in the T_Mid_-pH_Hi_ condition compared with the T_Hi_-pH_Norm_, T_Hi_-pH_Hi_, and T_Lo_-pH_Norm_ conditions (*p* < 0.05).

### 3.4. Salivary [NO_2_^−^]

**Figure 3:**
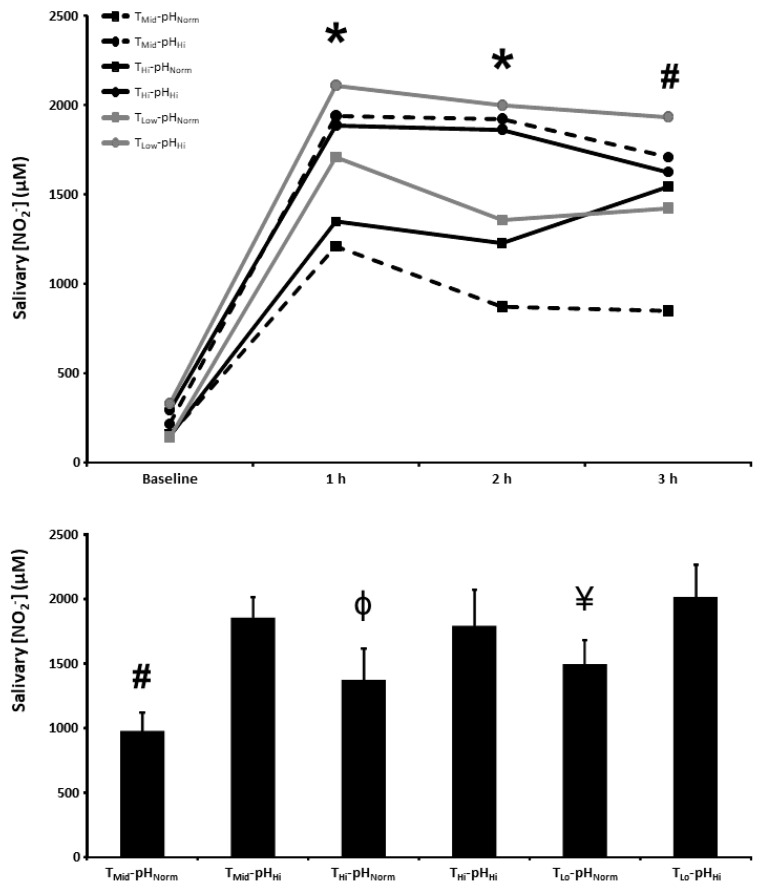
Salivary nitrite concentration ([NO_2_^−^]) time-course (upper panel) and the mean salivary [NO_2_^−^] at 1–3 h of the protocol (lower panel) after acute nitrate-rich beetroot juice ingestion in intermediate temperature−neutral pH (T_Mid_-pH_Norm_), intermediate temperature−high pH (T_Mid_-pH_Hi_), high temperature−neutral pH (T_Hi_-pH_Norm_), high temperature and pH (T_Hi_-pH_Hi_), low temperature−neutral pH (T_Lo_-pH_Norm_), and low temperature−high pH (T_Lo_-pH_Lo_) conditions. Salivary [NO_2_^−^] values across the protocol (upper panel) are expressed as group mean values with error bars omitted for clarity. The filled bars represent the group mean ± SEM responses in each experimental condition for mean salivary [NO_2_^−^] at 1–3 h of the protocol (lower panel). * indicates T_Mid_-pH_Norm_ lower than T_Mid_-pH_Hi_, T_Hi_-pH_Hi_, T_Lo_-pH_Norm_, and T_Lo_-pH_Hi_ (*p* < 0.05). # indicates T_Mid_-pH_Norm_ lower than T_Mid_-pH_Hi_, T_Hi_-pH_Norm_, T_Hi_-pH_Hi_, T_Lo_-pH_Norm_, and T_Lo_-pH_Hi_ (*p* < 0.05). Φ indicates lower than T_Hi_-pH_Hi_ and T_Lo_-pH_Hi_ (*p* < 0.05). ¥ indicates lower than T_Lo_-pH_Hi_ (*p* < 0.05).

The salivary [NO_2_^−^] at baseline and 1, 2, and 3 h post BR ingestion and the mean salivary [NO_2_^−^] 1–3 h post BR ingestion are presented in Figure 3. There were main effects for time (*p* < 0.001) and condition (*p* < 0.01), as well as a condition × time interaction effect (*p* < 0.05), for salivary [NO_2_^−^]. Salivary [NO_2_^−^] was higher 1 and 2 h post BR ingestion in the T_Mid_-pH_Hi_, T_Hi_-pH_Hi_, T_Lo_-pH_Norm_, and T_Lo_-pH_Hi_ conditions compared with the T_Mid_-pH_Norm_ condition (*p* < 0.05), and higher than the T_Mid_-pH_Norm_ condition in all of the other experimental conditions 3 h post BR ingestion (*p* < 0.05). There was a main effect for condition on the mean salivary [NO_2_^−^] 1–3 h post BR consumption (*p* < 0.01) with the mean salivary [NO_2_^−^] being higher than the T_Mid_-pH_Norm_ (976 ± 388 µM) condition in T_Mid_-pH_Hi_ (1855 ± 423 µM) and all other experimental conditions (*p* < 0.05), higher than the T_Hi_-pH_Norm_ (1371 ± 653 µM) condition in the T_Hi_-pH_Hi_ (1792 ± 741 µM) and T_Lo_-pH_Hi_ (2013 ± 662 µM) conditions (*p* < 0.05), and higher than the T_Lo_-pH_Norm_ (1495 ± 502 µM) condition in the T_Lo_-pH_Hi_ condition (*p* < 0.05).

### 3.5. Plasma [NO_3_^−^]

**Figure 4:**
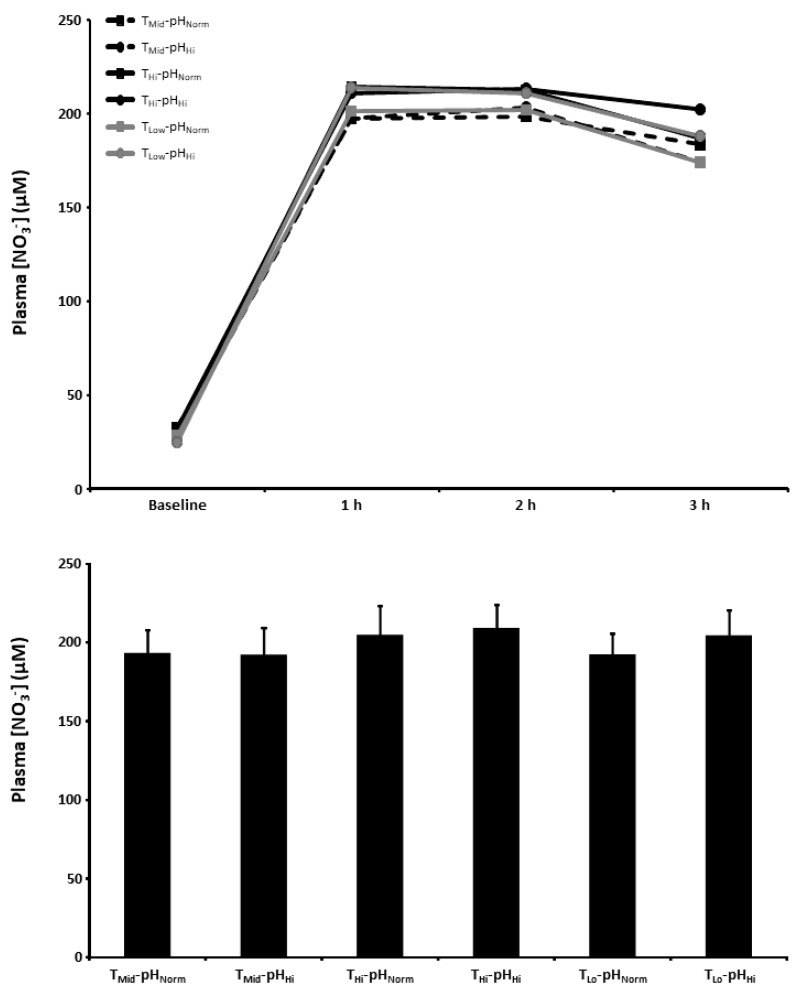
Plasma nitrate concentration ([NO_3_^−^]) time-course (upper panel) and the mean plasma [NO_3_^−^] at 1–3 h of the protocol (lower panel) after acute nitrate-rich beetroot juice ingestion in intermediate temperature−neutral pH (T_Mid_-pH_Norm_), intermediate temperature−..high pH (T_Mid_-pH_Hi_), high temperature−neutral pH (T_Hi_-pH_Norm_), high temperature and pH (T_Hi_-pH_Hi_), low temperature−neutral pH (T_Lo_-pH_Norm_), and low temperature and pH (T_Lo_-pH_Lo_) conditions. Plasma [NO_3_^−^] values across the protocol (upper panel) are expressed as group mean values with error bars omitted for clarity. The filled bars represent the group mean ± SEM responses in each experimental condition for mean plasma [NO_3_^−^] at 1–3 h of the protocol (lower panel).

The plasma [NO_3_^−^] at baseline and 1, 2, and 3 h post BR ingestion and the mean plasma [NO_3_^−^] 1–3 h post BR ingestion are presented in Figure 4. There was a main effect for time (*p* < 0.001) with plasma [NO_3_^−^] increasing above baseline in all experimental conditions. There were no between-condition differences in the plasma [NO_3_^−^] at 1, 2, and 3 h post BR ingestion or the mean plasma [NO_3_^−^] 1–3 h post BR ingestion (*p* > 0.05).

### 3.6. Plasma [NO_2_^−^]

**Figure 5:**
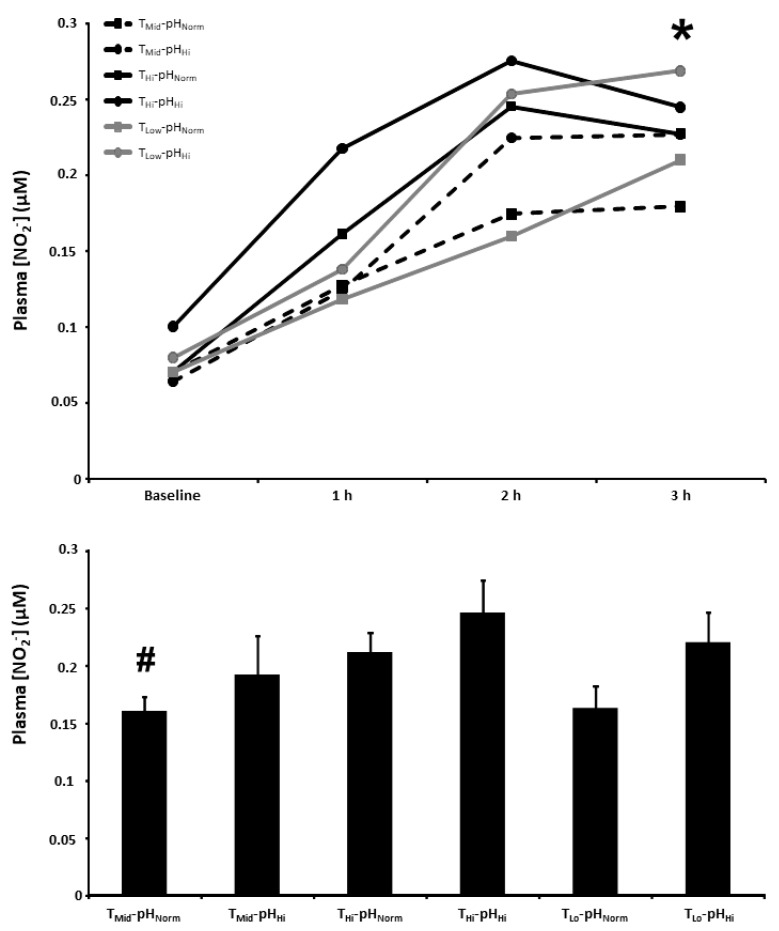
Plasma nitrite concentration ([NO_2_^−^]) time-course (upper panel) and the mean plasma [NO_2_^−^] at 1–3 h of the protocol (lower panel) after acute nitrate-rich beetroot juice ingestion in intermediate temperature−neutral pH (T_Mid_-pH_Norm_), intermediate temperature−high pH (T_Mid_-pH_Hi_), high temperature−neutral pH (T_Hi_-pH_Norm_), high temperature and pH (T_Hi_-pH_Hi_), low temperature−neutral pH (T_Lo_-pH_Norm_), and low temperature and pH (T_Lo_-pH_Lo_) conditions. Plasma [NO_2_^−^] values across the protocol (upper panel) are expressed as group mean values with error bars omitted for clarity. The filled bars represent the group mean ± SEM responses in each experimental condition for mean plasma [NO_2_^−^] at 1–3 h of the protocol (lower panel). * indicates T_Mid_-pH_Norm_ lower than T_Mid_-pH_Hi_, T_Hi_-pH_Hi_, and T_Lo_-pH_Hi_ (*p* < 0.05). # indicates lower than T_Hi_-pH_Hi_ (*p* < 0.05).

The plasma [NO_2_^−^] at baseline and 1, 2, and 3 h post BR ingestion and the mean plasma [NO_2_^−^] 1–3 h post BR ingestion are presented in Figure 5. There were main effects for time (*p* < 0.001) and condition (*p* < 0.05). The plasma [NO_2_^−^] 3 h post BR ingestion was higher than the T_Mid_-pH_Norm_ (179 ± 65 nM) condition in the T_Mid_-pH_Hi_ (227 ± 93 nM), T_Hi_-pH_Hi_ (245 ± 99 nM), and T_Lo_-pH_Hi_ (268 ± 110 nM) conditions (*p* < 0.05), but not the T_Hi_-pH_Norm_ (227 ± 73 nM) and T_Lo_-pH_Norm_ (210 ± 87 nM) conditions (*p* > 0.05). There was a main effect for condition on the mean plasma [NO_2_^−^] 1–3 h post BR consumption (*p* < 0.01) with the mean plasma [NO_2_^−^] higher than the T_Mid_-pH_Norm_ (160 ± 34 nM) condition in the T_Hi_-pH_Hi_ (246 ± 75 nM) condition (*p* < 0.05), but not the T_Mid_-pH_Hi_ (192 ± 89 nM), T_Hi_-pH_Norm_ (211 ± 45 nM), T_Lo_-pH_Norm_ (163 ± 51 nM), or T_Lo_-pH_Hi_ (220 ± 71 nM) conditions (*p* > 0.05).

## 4. Discussion

In the present study, modulating oral cavity temperature and pH influenced oral NO_3_^−^ reduction, as reflected by the altered salivary [NO_2_^−^] after NO_3_^−^-rich BR ingestion. Specifically, compared with the standard conditions of oral temperature and pH, elevating or lowering oral temperature and increasing oral pH independently augmented mean salivary [NO_2_^−^] after ingesting the same oral NO_3_^−^ dose. However, the effects of oral temperature and pH were not additive, as the mean salivary [NO_2_^−^] after BR ingestion was increased at a given temperature with a higher pH, with no differences between the high pH conditions. Plasma [NO_2_^−^] 3 h post NO_3_^−^ ingestion was not significantly impacted by oral temperature, but was elevated at a given oral temperature when pH was increased, with no difference between the high pH conditions. Therefore, while the observation that mean salivary [NO_2_^−^] increased to a greater extent after NO_3_^−^ ingestion when oral temperature and pH were independently increased is consistent with our first experimental hypothesis, the lack of an additive effect of the increasing oral temperature and pH on salivary and plasma [NO_2_^−^] after NO_3_^−^ ingestion does not support our second experimental hypothesis. These original observations improve our understanding of the factors that can influence dietary NO_3_^−^ metabolism in humans, and suggest that oral pH appears to have a greater overall influence than oral temperature.

Consistent with numerous previous reports [7,8,15,22,23,24,25,29,30,31], acute NO_3_^−^ ingestion increased salivary and plasma [NO_3_^−^] and [NO_2_^−^]. There were no between-condition differences in plasma [NO_3_^−^] in the present study. The mean salivary [NO_3_^−^] 1–3 h post BR ingestion was lower in the T_Mid_-pH_Hi_ condition compared to the T_Hi_-pH_Norm_, T_Hi_-pH_Hi_ and T_Lo_-pH_Norm_ conditions. The mechanisms for this effect may be linked to the influence of temperature and pH, and the interaction between these variables, on the salivary flow rate [32,33]. In addition, a physiological increase in temperature could have increased the NO_3_^−^ reductase activity of the oral microbiome, leading to a lower salivary [NO_3_^−^] in the T_Hi_-pH_Norm_ and T_Hi_-pH_Hi_ conditions compared with the T_Mid_-pH_Hi_ condition. Moreover, the uptake of NO_3_^−^ into the salivary glands is linked to the content and activity of the NO_3_^−^/H^+^ cotransporter, sialin [6]. The activity of sialin is pH-dependent and increases as extracellular pH declines [6], which could account for the lower salivary [NO_3_^−^] in the T_Mid_-pH_Hi_ condition compared with the T_Hi_-pH_Norm_ and T_Lo_-pH_Norm_ conditions. It is likely that some combination of these factors contributed to the lower salivary [NO_3_^−^] post BR ingestion in the T_Mid_-pH_Hi_ condition compared with the T_Hi_-pH_Norm_, T_Hi_-pH_Hi_, and T_Lo_-pH_Norm_ conditions.

Compared with the T_Mid_-pH_Norm_ condition, the mean salivary [NO_2_^−^] 1–3 h post BR ingestion was higher in all of the remaining experimental conditions (T_Mid_-pH_Hi_, T_Hi_-pH_Norm_, T_Hi_-pH_Hi_, T_Lo_-pH_Norm_, and T_Lo_-pH_Hi_). The greater increase in salivary [NO_2_^−^] after BR ingestion in the current study in Temp_Hi_-pH_Norm_ compared with Temp_Mid_-pH_Norm_ is consistent with previous reports of enhanced oral NO_3_^−^ reduction in the summer compared with the autumn or winter [26,28]. Our findings extend these earlier observations as oral temperature was not directly determined, and other factors that could influence oral NO_3_^−^ reduction and could have changed between seasons were not assessed in these studies. The increase in salivary [NO_2_^−^] after BR when oral temperature is increased is likely to be mediated by an increased rate of oral NO_3_^−^ reduction by the commensal bacteria at a higher temperature. However, the increase in salivary [NO_2_^−^] after BR ingestion in the current study was also greater in T_Lo_-pH_Norm_ compared with T_Mid_-pH_Norm_. This effect may be linked to an enhanced salivary flow rate and altered salivary composition at a lower temperature [34]. These effects might have enabled greater delivery of the NO_3_^−^ substrate to the oral NO_3_^−^ reducing bacteria, as well as other potential molecules transported in the saliva that could aid NO_3_^−^ reduction to NO_2_^−^.

Salivary [NO_2_^−^] after BR ingestion was higher in T_Mid_-pH_Hi_ compared with T_Mid_-pH_Norm_. This observation is consistent with previous reports that a more alkaline pH can aid NO_3_^−^ reduction to NO_2_^−^ [26,27,28,35]. The mean salivary pH in the T_Mid_-pH_Hi_, T_Hi_-pH_Hi_, and T_Lo_-pH_Hi_ conditions in the present study was 7.8 ± 0.2 and close to the purported optimal of pH 8 for oral NO_3_^−^ reduction [26,27,28]. It is now clear that certain genera of the oral microflora, including *Actinomyces*, *Granulicatella*, *Haemophilus*, *Neisseria*, *Prevotella*, *Rothia,* and *Veillonella* catalyse the reduction of NO_3_^−^ to NO_2_^−^ [8,9,10]. There is evidence that acute [36,37] and short-term [10] NO_3_^−^ supplementation modulates the abundance of some bacterial species, with increases in *Neisseria* and *Rothia* and declines in *Prevotella* and *Veillonella* having been reported. Moreover, it has been reported that the abundance and/or NO_3_^−^ reductase activity of some of these bacteria, such as *Prevotella*, *Rothia,* and *Veillonella* is enhanced with a more alkaline pH [35,38], which may account for the higher salivary [NO_2_^−^] after BR ingestion when oral pH was elevated in the current study. In addition, it is clear that in more acidic conditions, there is increased protonation of NO_2_^−^ to nitrous acid, which is subsequently decomposed to NO and other reactive nitrogen intermediates [20]. Indeed, it has been reported that exposing NO_3_^−^ reducing bacteria to a more acidic pH leads to greater NO_2_^−^ reduction compared with a more alkaline pH [35]. Therefore, the greater salivary [NO_2_^−^] after BR ingestion when oral pH was increased in the current study may be linked to greater oral NO_3_^−^ reduction, via the increased abundance or NO_3_^−^ reductase activity of NO_3_^−^ reducing bacteria, and to a lower decomposition of the synthesised NO_2_^−^ in the oral cavity. While the increasing oral temperature and pH independently increased the mean salivary [NO_2_^−^] after BR ingestion, these effects were not additive or synergistic.

Following acute ingestion of BR, plasma [NO_2_^−^] typically peaks after 2.5–3 h, concomitant with peak reductions in blood pressure [12,39]. As such, our discussion of the plasma [NO_2_^−^] data is focused on the 3 h time point, which has greater potential physiological relevance compared with the mean plasma [NO_2_^−^] 1–3 h post BR ingestion. Although the increasing core temperature via hot water immersion can increase the plasma [NO_2_^−^] [40], local heating does not appear to have the same effect [41]. Consistent with this latter observation, local heating (or cooling) of the oral cavity did not impact plasma [NO_2_^−^] in the current study after BR ingestion. Conversely, compared with T_Mid_-pH_Norm_, plasma [NO_2_^−^] 3 h post BR ingestion was higher in T_Mid_-pH_Hi_, T_Hi_-pH_Hi_, and T_Lo_-pH_Hi_. Therefore, while increasing the oral temperature and pH can independently increase salivary [NO_2_^−^] post BR ingestion, the plasma [NO_2_^−^] post BR ingestion was not impacted by altering oral temperature but was elevated when increasing the oral pH. Collectively, these data suggest that manipulating the oral pH has a greater overall impact on dietary NO_3_^−^ metabolism than oral temperature.

The original observations presented in this study improve our understanding of some of the factors that impact dietary NO_3_^−^ metabolism in healthy adults. As improvements in cardiovascular health markers and exercise responses are positively associated with oral NO_3_^−^ reduction and the increase in plasma [NO_2_^−^] after NO_3_^−^ supplementation [12,15,16,17], our findings might have implications for the potential for BR supplementation to enhance vascular function and exercise performance. Our findings also have implications for the standardisation of practices in future studies assessing the effects of NO_3_^−^ supplementation on salivary and plasma [NO_2_^−^], as the former can be altered by oral temperature and pH, and the latter can be altered by oral pH. However, it is acknowledged that a major limitation of the current study is the small sample size, such that the preliminary findings presented in this pilot study require more comprehensive investigation in future studies. While participants were instructed to avoid NO_3_^−^-rich foods for 48 h prior to the testing sessions, the lack of dietary assessment to evaluate NO_3_^−^ intake prior to and during the experimental testing is a limitation of the current study. In addition, other aspects of oral physiology, such as salivary flow rate and the composition of the oral microbiome, were not measured and would have provided greater insight into the mechanisms responsible for our observations. Indeed, it is possible that altering the oral pH influenced the salivary flow rate and that nasal breathing with a closed mouth in the higher oral temperature conditions altered the oral cavity oxygen tension, which could have impacted the activity of the oral anaerobic NO_3_^−^ reducing bacteria. While breathing with an open mouth and only breathing through the mouth successfully lowered oral temperature, this also likely impacted mouth dryness, which could have influenced other aspects of oral physiology, such as the salivary flow rate. Moreover, although the temperature manipulations were effective at eliciting the desired directional modulation of oral temperature within participants, there was pronounced between-participant variability in the changes in oral temperature induced by the oral temperature manipulations in the current study. Therefore, further research is required to evaluate the effect of oral temperature and its interaction with oral pH on dietary NO_3_^−^ metabolism and its physiological effects. It should also be recognised that while increasing oral pH via mouth rinses may increase salivary and plasma [NO_2_^−^] after NO_3_^−^ supplementation, the consumption of beverages with a high pH has the potential to compromise some of the beneficial responses observed following NO_3_^−^ supplementation by increasing the gastric pH and altering the formation of reactive nitrogen intermediates in the stomach and their appearance in the plasma. Indeed, it has been reported that while increasing gastric pH had no influence on the plasma [NO_2_^−^], this attenuated plasma [S-nitrosothiols] and the lowering of blood pressure after NO_3_^−^ supplementation [42].

## 5. Conclusions

The present study indicates that independently increasing or lowering oral temperature or increasing oral pH significantly increased mean salivary [NO_2_^−^] after NO_3_^−^ supplementation. However, the increase in plasma [NO_2_^−^] 3 h after NO_3_^−^ supplementation was not impacted by altering the oral temperature, but was increased when the oral pH was elevated, irrespective of oral temperature. These observations contribute to understanding the factors that can influence dietary NO_3_^−^ metabolism in healthy humans and highlight the need to standardise oral temperature and pH when assessing salivary [NO_2_^−^], and oral pH when assessing plasma [NO_2_^−^] in NO_3_^−^ supplementation studies.

## Data Availability

Data are available from the corresponding author upon reasonable request.
